# Biocrust carbon exchange varies with crust type and time on Chihuahuan Desert gypsum soils

**DOI:** 10.3389/fmicb.2023.1128631

**Published:** 2023-05-10

**Authors:** Mikaela R. Hoellrich, Darren K. James, David Bustos, Anthony Darrouzet-Nardi, Louis S. Santiago, Nicole Pietrasiak

**Affiliations:** ^1^Plant and Environmental Sciences, New Mexico State University, Las Cruces, NM, United States; ^2^USDA-ARS Jornada Experimental Range, New Mexico State University, Las Cruces, NM, United States; ^3^US DOI White Sands National Park, Alamogordo, NM, United States; ^4^Biological Sciences, The University of Texas at El Paso, El Paso, TX, United States; ^5^Botany and Plant Sciences, University of California, Riverside, Riverside, CA, United States; ^6^School of Life Sciences, University of Nevada, Las Vegas, Las Vegas, NV, United States

**Keywords:** White Sands National Park, dryland, carbon fixation, cyanobacteria, *Peltula*, *Clavascidium*, desiccation recovery

## Abstract

**Introduction:**

In dryland systems, biological soil crusts (biocrusts) can occupy large areas of plant interspaces, where they fix carbon following rain. Although distinct biocrust types contain different dominant photoautotrophs, few studies to date have documented carbon exchange over time from various biocrust types. This is especially true for gypsum soils. Our objective was to assess the carbon exchange of biocrust types established at the world’s largest gypsum dune field at White Sands National Park.

**Methods:**

We sampled five different biocrust types from a sand sheet location in three different years and seasons (summer 2020, fall 2021, and winter 2022) for carbon exchange measurements in controlled lab conditions. Biocrusts were rehydrated to full saturation and light incubated for 30 min, 2, 6, 12, 24, and 36 h. Samples were then subject to a 12-point light regime with a LI-6400XT photosynthesis system to determine carbon exchange.

**Results:**

Biocrust carbon exchange values differed by biocrust type, by incubation time since wetting, and by date of field sampling. Lichens and mosses had higher gross and net carbon fixation rates than dark and light cyanobacterial crusts. High respiration rates were found after 0.5 h and 2 h incubation times as communities recovered from desiccation, leveling off after 6 h incubation. Net carbon fixation of all types increased with longer incubation time, primarily as a result of decreasing respiration, which suggests rapid recovery of biocrust photosynthesis across types. However, net carbon fixation rates varied from year to year, likely as a product of time since the last rain event and environmental conditions preceding collection, with moss crusts being most sensitive to environmental stress at our study sites.

**Discussion:**

Given the complexity of patterns discovered in our study, it is especially important to consider a multitude of factors when comparing biocrust carbon exchange rates across studies. Understanding the dynamics of biocrust carbon fixation in distinct crust types will enable greater precision of carbon cycling models and improved forecasting of impacts of global climate change on dryland carbon cycling and ecosystem functioning.

## 1. Introduction

In dryland systems, biological soil crusts (biocrusts) can occupy large areas of plant interspaces. Biocrusts are biological features formed at the soil surface by diverse communities of microbial organisms and cryptogams (Weber et al., 2022). They can be classified into different community types defined by their dominant photoautotroph (Pietrasiak et al., 2013). These biocrust types organize across a gradient of increasing structural complexity: from light cyanobacterial to dark cyanobacterial crusts, where most biomass exists within the topsoil matrix, to lichens and bryophyte crusts with distinct aboveground structures. The dominant photoautotroph dictates changes to the broader microbial community composition within the biocrust, resulting in different nutrient cycling profiles that reflect the differing biogeochemical abilities of the organisms present (Maier et al., 2018; Lan et al., 2021). Therefore, landscape to global level biogeochemical models benefit from investigations that profile biocrust types separately for quantities and qualities of their biogeochemical contributions.

One of the most notable biogeochemical contributions of biocrust cryptogams is their ability to perform photosynthesis. They can contribute significantly to primary productivity and are thus an important component to understanding dryland carbon flux (Steiner et al., 2023). In general, different biocrust types have been shown to have different carbon fixing capacities under optimal conditions, with lichens and mosses having the highest carbon fixing potentials (Housman et al., 2006; Grote et al., 2010; Maestre et al., 2013; Pietrasiak et al., 2013; Ladrón de Guevara et al., 2014; Miralles et al., 2018; Tamm et al., 2018). Despite these differences, only a few extensive field-based studies have been carried out that differentiate among biocrust types—an omission due in part to the generally mosaic-like makeup of biocrust cover, with many crust types often growing alongside each other in small areas (Housman et al., 2006; Maestre et al., 2013; Ladrón de Guevara et al., 2014; Miralles et al., 2018). Many studies obtain composite rates of different biocrust types when taking measurements, complicating comparisons of individual community dynamics (Housman et al., 2006; Grote et al., 2010; Dettweiler-Robinson et al., 2018).

The physiological adaptations of biocrust organisms to dryland environments have important implications for assessment of carbon exchange. Biocrust communities are poikilohydric, physiologically active only when water is available (Weber et al., 2022). Because biocrust microbes are in a desiccated state for extended periods until the next hydration event, recovery time is needed before the community can reestablish maximum photosynthetic capacity (Satoh et al., 2002; Graham et al., 2006; Proctor et al., 2007; Abed et al., 2014; Wu et al., 2017). Respiration is the first physiological process to recover after wetting, beginning within seconds to minutes of rehydration as cellular processes reestablish and photosynthetic pigments are resynthesized and repaired (Abed et al., 2014). For example, high rates of respiration were recorded during the first 6 to 8 h after rehydrating desiccation tolerant cyanobacteria *Nostoc commune* and *Nostoc flagelliforme* (Scherer et al., 1984). Photosynthetic re-activation can also be rapid. For example, in *N. commune*, photosynthesis was detected within 10 min of water addition with a return to half the peak photosynthetic capacity within 1 h (Satoh et al., 2002). Desiccation tolerant mosses, such as *Tortula princeps* (De Not.), and lichens, such as *Collema tenax,* were reported to have similar recovery rates (Graham et al., 2006; Proctor et al., 2007; Wu et al., 2017). The speed of dry down preceding desiccation and the length of the desiccation period itself can both impact the recovery rate observed in mosses due to damage accrued during these periods (Proctor et al., 2007; Munzi et al., 2019). Together, initially high respiration and delayed photosynthesis reestablishment mean that net carbon fixation rate will vary depending upon time since wetting and may differ among biocrust types. These timings make comparing biocrust carbon fixation rates across studies challenging when the time since rehydration before taking measurements may range anywhere from minutes to days (Housman et al., 2006; Grote et al., 2010; Maestre et al., 2013; Pietrasiak et al., 2013; Dettweiler-Robinson et al., 2018; Miralles et al., 2018; Tamm et al., 2018).

Soil chemical composition is an important factor controlling biocrust abundance, species composition, and diversity (Rosentreter and Belnap, 2003; Bowker et al., 2006, 2016; Bowker and Belnap, 2008; Pietrasiak et al., 2011). In particular, biocrusts have often been observed to be especially dominant on gypsum soils (Rosentreter and Belnap, 2003; Bowker et al., 2016). Despite the importance of this soil type to biocrust ecology, few comparative studies have investigated the physiology of carbon fixation among the many biocrust types found on these soils. Two studies were carried out specifically on gypsum rich soils in Spain, where recorded maximum net carbon fixation rates did not exceed 1 μmol CO_2_ m^−2^ s^−1^ (Maestre et al., 2013; Miralles et al., 2018). To the best of our knowledge, only one lab-based study has examined carbon exchange by gypsiferous biocrust types. Raggio et al. (2014) investigated four different biocrust lichens with maximum net carbon fixation rates ranging from 1.95–2.85 μmol CO_2_ m^−2^ s^−1^ and one moss crust (2.27 μmol CO_2_ m^−2^ s^−1^). Perhaps, one of the best available sites to undertake such a study are the gypsum-rich soils associated with the extensive dunefields of White Sands National Park in New Mexico, USA. At this site, biocrust cover can be as high as 81%, of which up to 34.3% comprised lichen cover (Hoellrich, 2021). Here, we aim to add to the knowledge of carbon fixation physiology of these biocrust communities.

In this study, we investigated the extent and variability of carbon exchange of five different biocrust types (light cyanobacterial, dark cyanobacterial, cyanolichen, chlorolichen, and moss dominated crust) commonly found at White Sands National Park. Specifically, we collected crust specimens from the same location at three different time points (summer 2020, fall 2021, and winter 2022) and assessed respiration, carbon uptake, and net carbon fixation rates in controlled lab conditions. We asked the following questions: (Q1): Do biocrust carbon fixation rates differ by crust types? (Q2) How does the net fixation rate change across types with time since rehydration? (Q3) Do biocrust carbon fixation rates differ depending on time since the last rain event? If biocrust type is a good predictor of carbon fixing capacity, we would expect to observe significant differences in the carbon exchange rates of different biocrust types. Also, if biocrust communities require a recovery period before maximum carbon fixation can resume, then biocrust net fixation would increase with incubation time before measurement. Lastly, if biocrust carbon fixation differs by rain seasonality, then we would expect significant differences between the rates in the different sampling periods. In examining these questions, we will gain a better understanding of the physiological strategies of these biocrust organisms, gain valuable parameters for assessing the contributions of these organisms to overall ecosystem function, and make methodological recommendations for how to assess and model biocrust carbon exchange rates on local to global scales.

## 2. Materials and methods

### 2.1. Study area

**Figure 1:**
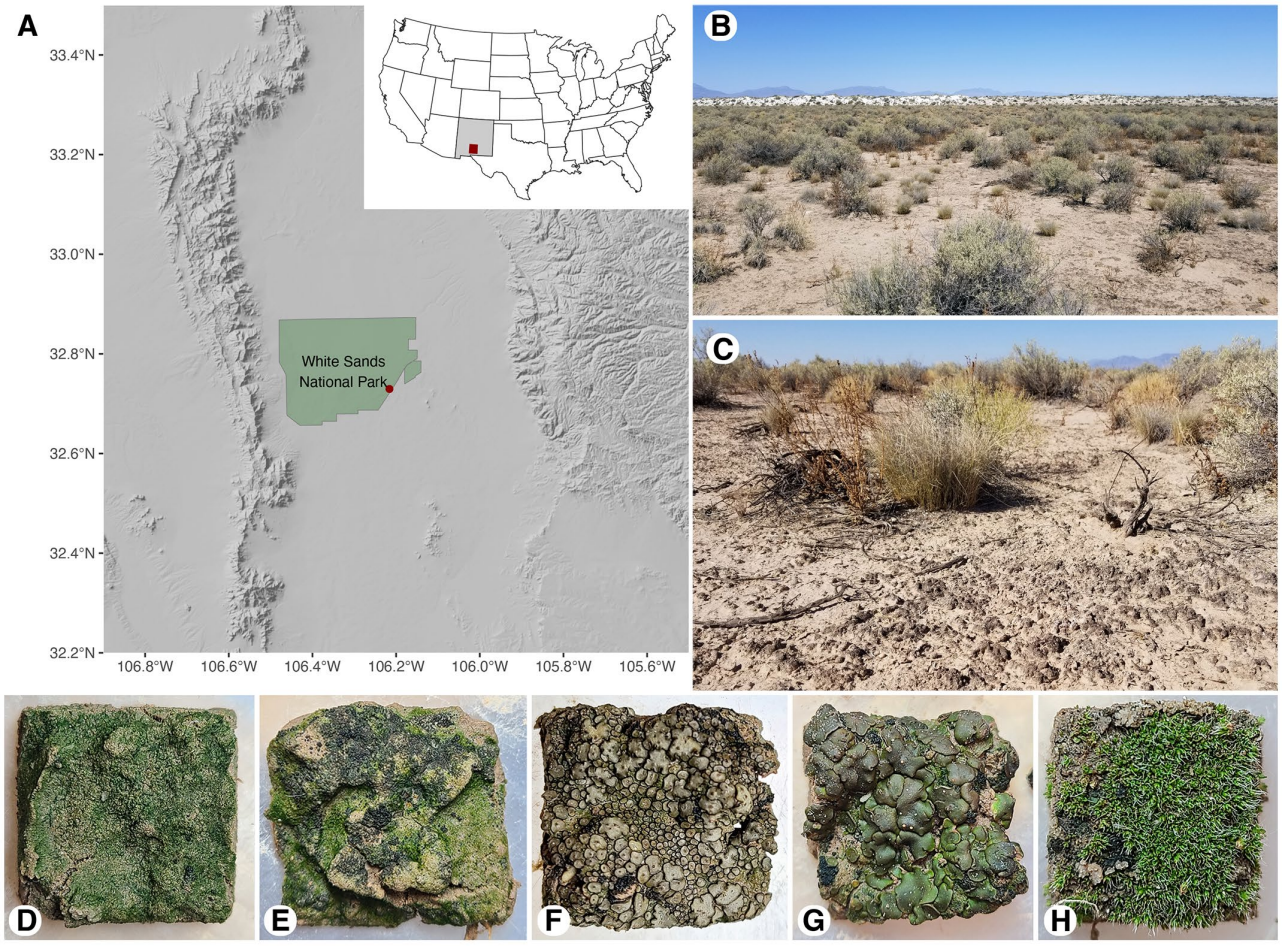
Map of White Sands National Park sampling location **(A)**, landscape view of sand sheet in 2020 **(B)**, site ground cover in 2020 **(C)**, biocrust types in hydrated state: light cyanobacterial **(D)**, dark cyanobacterial **(E)**, *Peltula* sp. cyanolichen **(F)**, *Clavascidium* sp. chlorolichen **(G)**, and moss dominated crust **(H)**.

A 45 × 60 m gypsum sand sheet area was sampled in three different months of three successive years at White Sands National Park, New Mexico, USA (Figures 1A–C; Supplementary Table 1). The first sampling occurred in July 2020 (summer), the second in September 2021 (fall), and the third in March 2022 (winter). Sampling time was chosen to represent different seasonal time points with different precipitation conditions. Summer 2020 was sampled before the start of the monsoon rains, fall 2021 after the majority of the monsoon rains occurred of the monsoon rains, and winter 2022 subject to the less substantial winter precipitation. Rain is the major form of precipitation in the Chihuahuan Desert. Pre-sampling rain information can be found in Table 1, long-term humidity data can be found in Supplementary Table 2, and long-term temperature and precipitation data in Supplementary Figure 1 (data collected from National Weather Service, 2023). Fog and dew can provide additional moisture to activate the metabolisms of biocrust organisms. Nearby park weather stations currently do not track instances of dew and fog. However, 17.24 km from our study site at the Holloman Air Force Base weather station (Horel et al., 2002), only nine instances of fog or mist were detected across the 2020–2022 period, none of which occurred in the periods of “no rain” before our sampling. Additionally, temperature dipped lower than the dew point only four times in this site in 2020–2022. Thus, our assumption is that these occurrences are rare. However, hourly maximum and minimum temperatures were not collected, so there may have been more examples that were not tracked. Currently there is no absolutely reliable way to assess dew contributions to hydration for biocrusts within White Sands National Park.

**Table 1:** Pre-sampling rain data for the gypsum sand sheet area investigated at White Sands National Park.

Season	Last precip (days)	Last precip (cm)	Last 60 days (cm)	Number of precip events
Summer 2020	18	0.28	1.45	6
Fall 2021	1	0.08	13.08	17
Winter 2022	34	0.33	1.35	4

*Last Precip (days)* indicates the number of days since the last precipitation event before sampling, *Last Precip (cm)* indicates the amount of rain at the last rain event, *Last 60 Days (cm)* indicates the amount of rain in the last 60 days before sampling, and *Number of Precip Events* indicates the number of rain events in the last 60 days.

A biocrust sample for carbon exchange measurement was defined as the soil surface aggregate (approximately 5 × 5 × 1 cm) that stays intact by its own aggregate strength during collection and has visible biofilaments. A pallet knife was used to extract samples, which were gently wrapped in paper towels and placed in dry paper cups for laboratory storage (no longer than 3 months at room temperature) and subsequent measurements. The sampling design for 2020 differed slightly from 2021 to 2022 in order to make assessments with higher replication and to understand if there was a significant change in carbon flux after 24 h. In 2020, we collected 5 biocrust functional types (light cyanobacterial, dark cyanobacterial, *Peltula* sp. cyanolichen, *Clavascidium* sp. chlorolichen, moss dominated crust with Pottiaceae spp.; Figures 1D–H) for each of five incubation times at a replication of five per type and incubation time (125 total number of samples collected and analyzed that year). Biocrust sampling was done along two 30 m transects crossing in an “X” at 15 m (Supplementary Table 1). We systematically collected one representative specimen of each biocrust for each incubation condition alongside a 1.6 × 10.55 m area of each transect line. For 2021 and 2022 we again established two intercrossing 30 m transects similar to 2020 but in a different quadrant of the sand sheet area (Supplementary Table 1). One of each replicate for each incubation condition was collected from a 1 × 15 m area. We increased the replication to 10 per type and investigated six incubation times (300 total samples).

### 2.2. Laboratory carbon exchange assessment

Carbon exchange measurements were performed under controlled laboratory conditions. Biocrusts were rehydrated to full saturation (the amount of water held by the soil without overflowing, characterized by water glistening at the surface of the sample) with reverse osmosis purified water. Samples were then cut into 1.7 × 1.6 cm rectangles with a tin clay cutter and light incubated at room temperature (PPFD 60–80 μmol m^−2^ s^−1^, measured with a Model MQ-200 Quantum meter, Apogee Instruments Inc., UT) for 30 min, 2, 6, 12, 24, or 36 h. Biocrust samples were not reused for multiple time intervals. To minimize potential confounding effects brought on by up to 3 months of storage after sampling as well as instrument variability, samples were systematically measured in a sequence of light cyanobacterial, dark cyanobacterial, cyanolichen, chlorolichen, and moss dominated at each time interval from 30 min, 2, 6, 12, 24, and 36 h. This pattern was followed for each replicate until all measurements were taken. For 2020, samples were rewet to saturation every 6 h as well as 30 min before taking carbon reading. When inserting into the machine samples were no longer glistening at the surface, although in 2020 it was noticed that for a small number of samples with higher clay content, water inhibition was occurring in the first few data points. For this reason, the last watering event for the 2021 and 2022 series was changed from half an hour to two hours before taking measurements. Samples were subjected to a 12-point light regime (PPFD: 0, 25, 50, 100, 150, 300, 500, 750, 1,000, 1,250, 1,600, and 2000 μmol m^−2^ s^−1^) with a LI-6400XT photosynthesis system. The DRIERITE desiccant was set to full scrub to remove as much water from the air as possible and relative humidity was monitored to avoid a high humidity alert and condensation within the machine. Across the 725 measurements taken for this study, the highest humidity readings were two at 85% and one 86%, both of which came from 2020 before the protocol was changed to wait 2 h after rehydration. The CO_2_ mixer was set to 400 μmol CO_2_ m^−2^ s^−1^, flow was set to 300 μmol CO_2_ m^−2^ s^−1^, and carbon fixation was determined under ambient temperature generated in the light chamber (~26.26 ± 2.8°C).

### 2.3. Data analysis

All data were analyzed in R version 4.2.1 (R Core Team, 2022) and R studio version 2022.07.1+554 (RStudio Team, 2022). The respiration rate was taken as the first value on the light response curve, collected as a negative value at PPFD 0 μmol m^−2^ s^−1^ and multiplied by -1 to get a positive value. The net fixation value was the maximum positive net fixation rate reached for each light response curve. The gross carbon fixation value was calculated as the difference between the value at PPFD 0 μmol m^−2^ s^−1^ and the net fixation value. From the 2022 series, replicate five of light cyanobacterial crust incubated for 36 h was excluded from analysis because the rate at zero was over zero, which is indicative of a mechanical error.

The mean and standard error for gross carbon fixation, respiration, and net fixation were computed using the *dplyr* package’s (Wickham et al., 2022) arrange() function, grouping by season, type, and time. From the *Car* Package (Fox and Weisberg, 2019), the Anova() function was used to test for significant differences between groups. A three-way, type III ANOVA (analysis of variance) was run for gross carbon fixation, respiration, and net fixation, testing for significance between biocrust type, incubation time, and season of collection as fixed factors, including all interactions between variables. The 36 h interval was excluded from ANOVA testing as this time interval was not performed in 2020. From the *emmeans* package (Lenth, 2022), the emmeans() function was used to find pairwise mean comparisons *via* Tukey test within the groups. Additionally, the lm() function from the *stats* package was used to create linear models for AICc-based model comparison (R Core Team, 2022). Linear models were created to account for all factors and interactions tested within the three-way ANOVAs. The aictab() function from the *AICcmodavg* package was used to test which model was best fitted to the data set (Mazerolle, 2020). Data files are published to EDI Data Portal under the doi:10.6073/pasta/c6ffd88dc80df1ed1ec32ccdc477ac61.

## 3. Results

### 3.1. AIC model testing

AIC (Akaike information criterion) model testing indicated that for gross carbon fixation and respiration, biocrust type was the best fitting single fixed factor explaining most of the data variability, followed by sampling season (Season) and then incubation time (Time) (Table 2). For net carbon fixation, time was the best fitting fixed factor for explaining variability, followed by season, then type. The best model for explaining gross carbon fixation, respiration, and net carbon fixation overall was the Season*Type*Time interaction, followed by Season*Type for gross carbon fixation and respiration, and Season*Time for net fixation (Table 2).

**Table 2:** Results of AIC comparison of linear models for gross carbon fixation, respiration, and net carbon fixation, where *Model* indicates the factors of the linear model, and *AICc* indicates the second order Akaike Information Criterion, where the smallest value is the best fit and models are ordered from best to worst fit.

Gross fixation		Respiration		Net fixation	
Model	AICc	Model	AICc	Model	AICc
Season*Type*Time	2498.34	Season*Type*Time	2163.01	Season*Type*Time	2660.55
Season*Type	2516.31	Season*Type	2266.78	Season*Time	2801.24
Time*Type	2591.48	Time*Type	2300.91	Season*Type	2817.54
Type	2608.25	Type	2394.39	Time*Type	2848.46
Season*Time	2950.59	Season*Time	2456.05	Time	2916.2
Season	2956.91	Season	2526.29	Season	2930.15
Time	2971.78	Time	2537.55	Type	2956.19
null	2980.77	null	2604.54	null	3016.81

### 3.2. ANOVA testing

Biocrust type, incubation time, season of collection, and all interactions were significant in accounting for variability of rates for gross carbon fixation, and net carbon fixation (Table 3). However, for gross carbon fixation, and net fixation Time:Type and Time:Season:Type models were only significant when mosses were included in the data set. Additionally, for respiration the Time:Season and Time:Type:Season models were not significant even when mosses were included.

**Table 3:** Table of ANOVA results for gross fixation, respiration, and net fixation across all three seasons, where df is degrees of freedom and *Pr(>F)* is value of *p*.

		Gross fixation	Respiration	Net fixation
Source	df	*Pr(>F)*	*Pr(>F)*	*Pr(>F)*
(Intercept)	1	<0.001	<0.001	<0.001
Time	4	<0.001	<0.001	<0.001
Type	4	<0.001	<0.001	<0.001
Season	2	<0.001	<0.001	<0.001
Time:Type	16	0.001	0.001	<0.001
Time:Season	8	<0.001	0.056	<0.001
Type:Season	8	<0.001	<0.001	<0.001
Time:Type:Season	32	0.018	0.54	0.01

**Figure 2:**
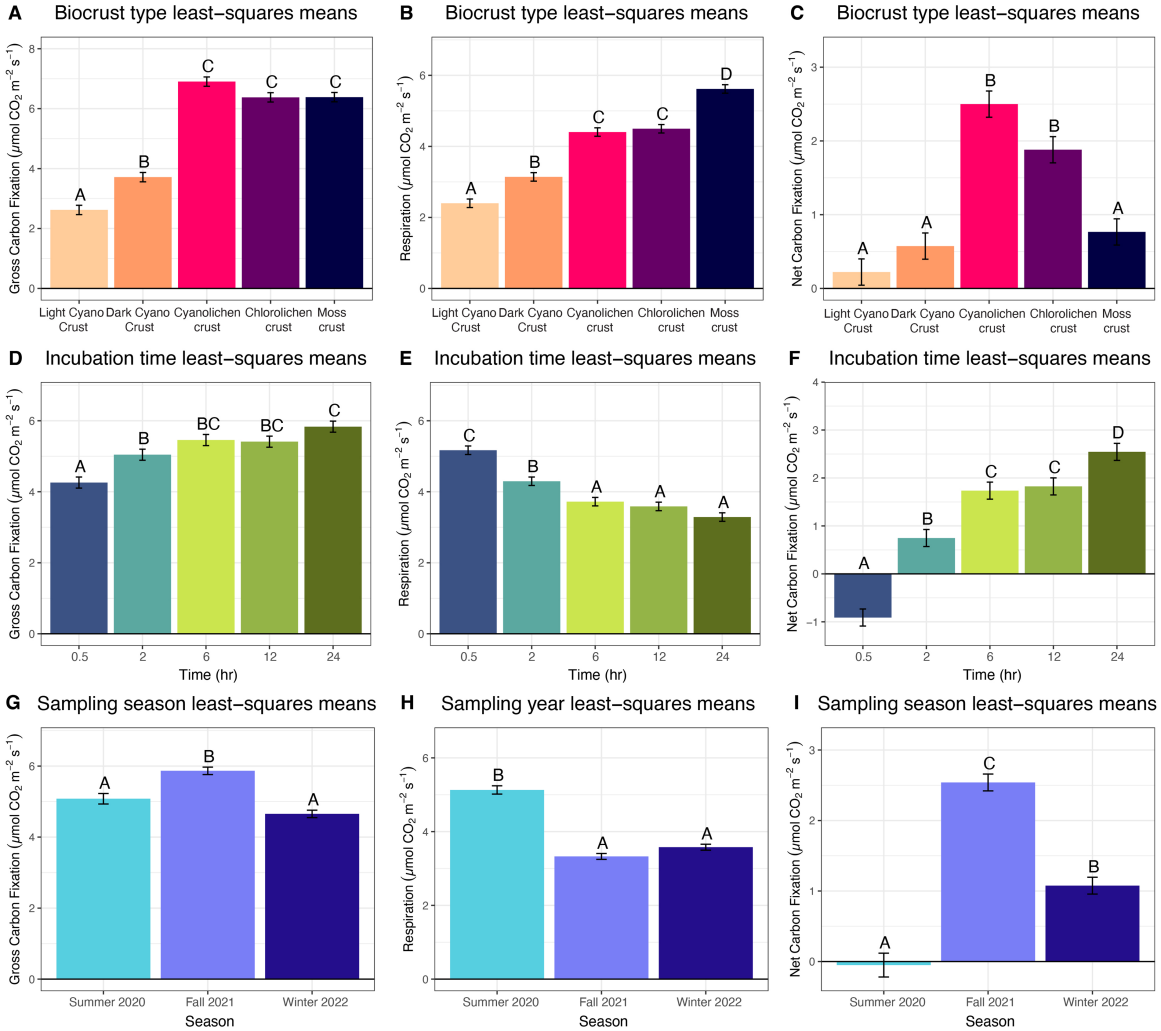
Estimated marginal means of main effects for biocrust gross carbon fixation, respiration and net carbon fixation at White Sands National Park, Chihuahuan Desert. **(A–C)** Biocrust type least square means averaged over season and time. **(D–F)** Incubation time least square means averaged over biocrust type and sampling time. **(G–I)** Sampling season least square means averaged over type and incubation time.

From the main effects, notwithstanding interactions, within type as a factor, dark cyanobacterial crusts had a gross carbon fixation rate significantly higher than light cyanobacterial. However, both cyanobacterial crusts had lower gross carbon fixation rates than lichen and moss crusts (Figure 2A). The respiration rate increased significantly from light cyanobacterial, to dark cyanobacterial, to both lichen types, and then to moss (Figure 2B). Net carbon fixation was significantly highest in cyanolichen and chlorolichen compared to all other crust types (Figure 2C).

Using incubation time as a main effect, gross carbon fixation generally increased over time while respiration decreased. Specifically, gross carbon fixation rates were significantly lower at 0.5 h incubation than at 2, 6, 12, and 24 h incubation, and rates at 2 h were also significantly lower than after 24 h incubation (Figure 2D). Respiration was significantly higher at 0.5 h than all other incubation times, and respiration at 2 h was significantly higher than at 6, 12, and 24 h (Figure 2E). Due to these gross carbon fixation and respiration relationships net carbon fixation increased significantly from rates measured at 0.5 h, to those at 2 h, and to values at 6 h and 12 h, and finally to those at 24 h (Figure 2F).

Analyzing the season as a main effect, gross carbon fixation was greatest in fall 2021, intermediate in summer 2020, and lowest in winter 2022, though there was no significant difference between summer 2020 and winter 2022 (Figure 2G). Respiration was significantly higher in summer 2020 than in fall 2021 and winter 2022 (Figure 2H). Net carbon fixation was highest in fall 2021, followed by winter 2022, and lowest in summer 2020 (Figure 2I).

**Figure 3:**
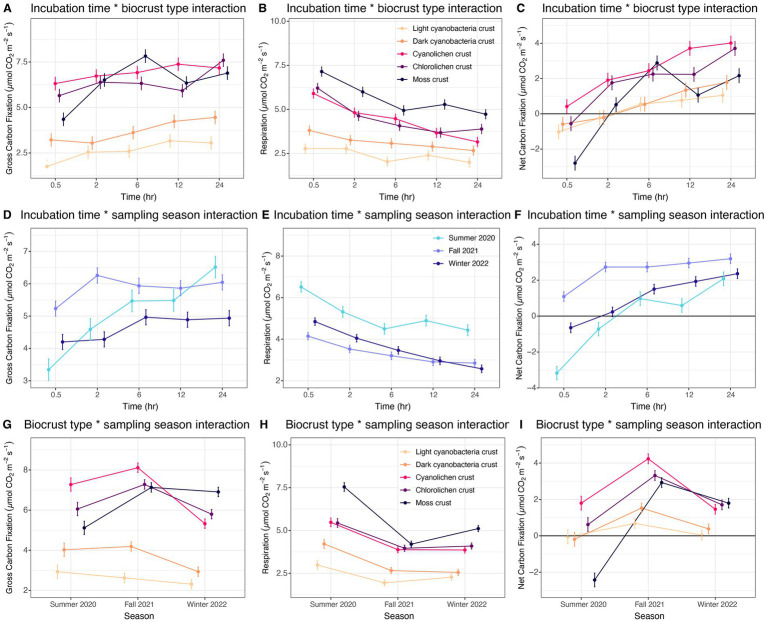
Estimated marginal means for two-way interactions for biocrust gross carbon fixation, respiration and net carbon fixation at White Sands National Park, Chihuahuan Desert. **(A–C)** Incubation time by biocrust type interaction averaged over season. **(D–F)** Incubation time by sampling season interaction averaged over biocrust type. **(G–I)**. Biocrust type by sampling season interaction averaged over incubation time.

Next, we explored patterns in the two-way interactions, analyzing biocrust type across incubation time. For gross carbon fixation, only moss crusts were observed to have a significant increase in the rate across incubation times (Figure 3A). Respiration significantly decreased across time intervals for moss, cyanolichen and chlorolichen crusts (Figure 3B). There was a decreasing trend in cyanobacterial crusts, though it was not significant. Net carbon fixation significantly increased for all crust types with incubation time, where moss had the highest increase, followed by cyanolichen, chlorolichen, dark cyanobacterial, and light cyanobacterial crusts (Figure 3C).

The interaction between time and season showed that for gross carbon fixation, only summer 2020 had a significant increase in rate across the time intervals (Figure 3D). Respiration significantly decreased across all seasons with the decrease in summer 2020 being largest and winter 2022 being second largest (Figure 3E). Net fixation significantly increased with time for all seasons with the largest increase being in summer 2020, followed by winter 2022 and fall 2021 (Figure 3F).

Across the interaction of biocrust type and season, cyanolichen crusts had the most fluctuation in gross carbon fixation rate across sampling dates followed by chlorolichen, moss, dark cyanobacterial, and light cyanobacterial crusts (Figure 3G). Moss crusts had the most fluctuation in respiration rate across the seasons followed by dark cyanobacterial crusts, light cyanobacterial crusts, then both lichen types (Figure 3H). Moss had the most fluctuation in net carbon fixation rates across the seasons, but in this case, it was followed by cyanolichen, chlorolichen, dark cyanobacterial, and light cyanobacterial crusts (Figure 3I).

**Figure 4:**
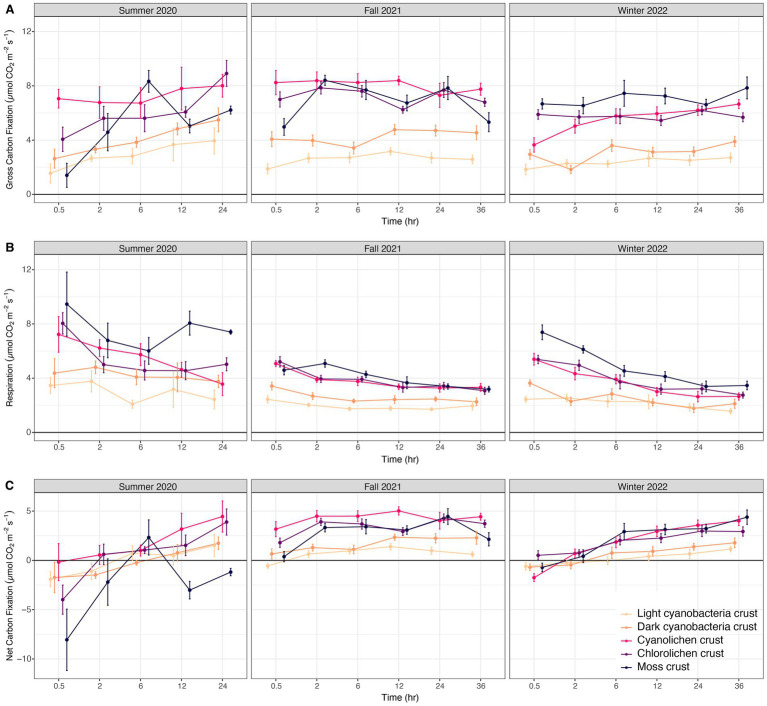
Biocrust gross carbon fixation **(A)**, respiration **(B)**, and net carbon fixation **(C)** at White Sands National Park, Chihuahuan Desert in sampling year Summer 2020, Fall 2021, and Winter 2022, after a set incubation time.

In the three-way interactions conducted for all three carbon exchange processes (Supplementary Table 3), gross carbon fixation significantly increased with incubation time in mosses in summer 2020 and fall 2021, in cyanolichen in winter 2022, and in chlorolichen in summer 2020. No crust types significantly differed from themselves over different seasons (Figure 4A). Gross carbon fixation was significantly lower in light cyanobacterial crusts than all other types for all seasons (except dark cyanobacterial crusts). Dark cyanobacterial cyanobacterial was not significantly lower than all other crust types. Respiration in cyanobacterial crusts did not significantly decrease over incubation time or across the three seasons (Figure 4B). Moss and lichen crusts had higher respiration than cyanobacterial crusts for all seasons. Net carbon fixation significantly increased with time for cyanolichen crust in fall 2021 and winter 2022, versus in chlorolichen and moss crusts for all seasons (Figure 4C). Net carbon fixation in moss was significantly lower at 0.5h, 12h, and 24h in summer 2020 than in fall 2021 and winter 2022. Net carbon fixation of light cyanobacterial crust was significantly lower than cyanolichen, chlorolichen and moss crusts in fall 2021 and winter 2022 at 24hr.

### 3.3. Maximum net fixation rates

Peak net fixation was reached at different incubation times between sampling seasons (Table 4). In winter 2020 most maximum rates were reached after 24 h, in fall 2021 after 12 h, and in winter 2022 after 36 h. Exceptions to this pattern occurred in moss crusts (peak values at 6 h incubation in summer 2020 and at 24 h in fall 2021) and chlorolichen crusts (peak values at 24 h in both 2021 and 2022). The highest net fixation rates for all crust types were found in fall 2021 (Table 4), except for light cyanobacterial crusts where the highest rate occured in summer 2020. Cyanolichen crusts had the highest net fixation rate for summer 2020 and fall 2021, versus the second highest rate in winter 2022 after moss crust (Table 4).

**Table 4:** Highest rate of net fixation for each biocrust type where *LCC* is light cyanobacterial crust, *DCC* is dark cyanobacterial crust, *CYL* is cyanolichen crust, *CHL* is chlorolichen crust, *MOS* is moss dominated crust.

Season	Type	Time (hr)	Net (μmol m^−2^ s^−1^)
Summer 2020	LCC	Twenty-four	1.51 ± 1.11
Fall 2021	LCC	Twelve	1.39 ± 0.33
Winter 2022	LCC	Thirty-six	1.15 ± 0.27
Summer 2020	DCC	Twenty-four	1.74 ± 0.63
Fall 2021	DCC	Twelve	2.35 ± 0.31
Winter 2022	DCC	Thirty-six	1.78 ± 0.5
Summer 2020	CYL	Twenty-four	4.45 ± 1.58
Fall 2021	CYL	Twelve	5.02 ± 0.42
Winter 2022	CYL	Thirty-six	4.01 ± 0.47
Summer 2020	CHL	Twenty-four	3.89 ± 1.32
Fall 2021	CHL	Twenty-four	4.27 ± 0.44
Winter 2022	CHL	Twenty-four	2.97 ± 0.55
Summer 2020	MOS	Six	2.32 ± 1.78
Fall 2021	MOS	Twenty-four	4.45 ± 0.8
Winter 2022	MOS	Thirty-six	4.39 ± 0.73

## 4. Discussion

### 4.1. Biocrust carbon fixation rates differed by biocrust types

Biocrust type (light cyanobacterial, dark cyanobacterial, *Peltula* sp. cyanolichen, *Clavascidium* sp. chlorolichen, and moss dominated crust) was the most important single factor in explaining rate variability for gross carbon fixation and respiration. While not every biocrust type was significantly different from every other type at each time point in a single season, both lichen crust types and moss crusts had significantly higher rates of gross carbon fixation and respiration than either cyanobacterial crust type. Across multiple studies higher rates of gross fixation in lichens and mosses have been found in comparison to cyanobacterial crusts (Housman et al., 2006; Grote et al., 2010; Pietrasiak et al., 2013; Ouyang et al., 2017; Dettweiler-Robinson et al., 2018; Miralles et al., 2018; Tamm et al., 2018). Lichen and moss crusts are more structurally complex compared to light and dark cyanobacterial crust, and thus associated higher lichen and moss crust gross carbon fixation and respiration rates may be explained by greater chlorophyll content and biomass, as has been observed in other studies (Ouyang et al., 2017; Maier et al., 2018; Román et al., 2019; Hoellrich 2021; Lan et al., 2021).

Differences in respiration rates may also be a consequence of the physiological differences in the types of organisms found in specific biocrust types. Different rates of respiration have been found in the comparison of moss, lichen, and cyanobacteria species all of which have different constraints on respiration (De Nobel et al., 1998; Sundberg et al., 1999; Waite and Sack, 2010). For net fixation, while not significantly different at every data point, cyanobacterial crusts had lower rates than lichens and mosses, except in summer 2020, where moss net fixation was notably low. Because of the high respiration rates in combination with a low gross carbon fixation rate at 0.5h, in summer 2020 the moss crusts’ net fixation was significantly lower than lichen crust net fixation. The high respiration and low gross carbon fixation rates at 0.5h in summer 2020 detected in moss crusts are likely indicative of a higher amount of environmental stress and/or damage accrued during their last preceding dehydration episode and perhaps also during the intervening period of time before sampling.

In comparison to other studies, the highest rates of net fixation found for each biocrust type in our study (Table 4) were within the standard error of other published light cyanobacterial crust rates (Grote et al., 2010; Ouyang et al., 2017), while values reported by other authors were higher than ours for cyanolichen and chlorolichen crusts (Grote et al., 2010; Ouyang et al., 2017; Tamm et al., 2018). Moss crust net fixation rates in our study were higher than in Tamm et al. (2018), but lower than in Ouyang et al. (2017). These variations in rates may be attributed to optimal pre-measurement conditions and/or physiological differences in different lichens and mosses from different locations. All of the maximum photosynthesis measurements observed in this study were higher than the net fixation rates of field based studies on gypsum soil where net fixation did not exceed 1 μmol CO2 m^2^ s^−1^ and biocrust type was not considered as a factor (Maestre et al., 2013; Miralles et al., 2018). The maximum photosynthesis rates for lichens and moss crusts in our samples also exceeded those observed in the only other biocrust lab based carbon flux analysis on gypsum soil (1.95–2.85 μmol CO_2_ m^−2^ s^−1^ for lichens and 2.27 μmol CO_2_ m^−2^ s^−1^ for moss; Raggio et al., 2014). Additional research is required to understand the extent of carbon exchange rate variability observed across existing studies and to investigate if there is a relationship between carbon exchange and the soil parent material.

### 4.2. Net carbon fixation rate increased with incubation time

Incubation time was the most important single factor for explaining variability in net carbon fixation data. Gross carbon fixation increased with time from 0.5 to 2 h and continued to rise from 2 to 6 h, changes thereafter being non-significant, indicating that the majority of recovery had occurred by the 6 h mark. However, when comparing across all crust types, significant increases were only observed in chlorolichen crusts in summer 2020, and in moss crusts in summer 2020 and fall 2021. Any increase observed in cyanobacterial crusts was not significant. Also, only summer 2020 showed a significant increase with time in gross carbon fixation when averaging across all types. The lack of significant increases indicates that carbon fixing capacity rapidly recovers in all biocrust types, as was observed in other desiccation tolerant cyanobacteria, lichens, and mosses (Graham et al., 2006; Proctor et al., 2007; Wu et al., 2017).

Respiration significantly decreased up to 6 h, after that, the differences were not significant. The decrease in respiration with incubation time in lichen and moss crusts was seen across all seasons, while a decrease in cyanobacterial crust respiration was observable albeit not significant. This decline of respiration in the first 6–8 h of rehydration matches the response seen in other desiccation tolerant photosynthesizers (Scherer et al., 1984; Lange et al., 1992). Consequently, as gross carbon fixation increased and, more importantly, respiration decreased, net fixation significantly increased with time, the largest step occurring between 0.5 h and 2 h. All crust types except dark cyanobacterial crust in summer 2020 reached positive net fixation rates by 6 h. This suggests rapid recovery from desiccation allowing for higher rates of net fixation to take place. This rapid recovery is consistent with the organisms’ need to use water effectively during warm, wet monsoon rains on rapidly draining soils. It may also be relevant to why lower net fixation rates are recorded during summer months in field studies. If summer rain events are lower in volume or the surface soil water evaporates more rapidly in the heat, then there will be less time for desiccation recovery to take place, thus leading to lower net fixation values. Additionally, the activation of biocrusts by fog and dew has been observed in biocrusts across multiple deserts (Kidron et al., 2002; Prado and Sancho, 2007; Delgado-Baquerizo et al., 2013; Raggio et al., 2014; Büdel et al., 2018). If there are periods of the year at White Sands National Park where sufficient fog and/or dew occur to activate biocrusts in ways that do result in net increase of carbohydrate reserves, then that small window of activity may help cellularly prepare for high volume rain events to follow later. In that case, a net positive carbon fixation rate would be more rapidly achieved during such rain events. While fog and high relative humidity are not generally an aspect of life in the northern Chihuahuan Desert (Supplementary Table 2), there are currently no monitoring stations at White Sands National Park tracking the presence/absence of dew. Future studies would benefit from such a station.

### 4.3. Carbon fixation rates differed depending on sampling time during the year

Season was the second most important single variable explaining variability for gross carbon fixation, respiration, and net carbon fixation. From field-based studies, seasonal effects have been found to affect biocrust net carbon flux (Deane-Coe et al., 2012; Maestre et al., 2013). Positive net fixation has most often been observed in fall and winter months in contrast to net negative fluxes recorded in summer months (Deane-Coe et al., 2012; Maestre et al., 2013; Miralles et al., 2018) and was associated with higher water availability for a long period of time in mild temperatures, fitting with the findings of this study.

We found that gross carbon fixation rates were highest in fall 2021, the season with the most rain immediately before sampling, followed by summer 2020 which had the 2nd closest rain event before sampling, and winter 2022 which had the longest time since rain event. However, the difference between summer 2020 and winter 2022 was not significant. Delayed recovery of photosynthetic activity has been observed in biocrusts, and in lichens and cyanobacteria isolated from biocrusts that were subjected to long desiccation periods during which essential photosynthetic components like chlorophyll degrade (Lange et al., 1992; Harel et al., 2004; Proctor et al., 2007; Kranner et al., 2008; Munzi et al., 2019). Deane-Coe et al. (2012) also directly linked higher rates of net carbon balance with interannual precipitation events in biocrust mosses. However, in our study respiration was significantly higher in summer 2020 than in fall 2021 and winter 2022. This may be due partly to the change in methods (2020 samples were all watered 0.5 h before all measurements, while the other seasons were watered 2 h before), but that would not explain why the initial respiration rate at 0.5 h was so high in 2020. Therefore, it may more likely be a product of the environmental stress that samples were subjected to in the field during the summer in addition to severe drought conditions in the Chihuahuan Desert in summer 2020. This contrasts with our data from those samples collected in the fall of 2021 and winter of 2022.

High temperatures could cause a more rapid dehydration and rapid dehydration has been linked to larger respiration bursts upon rehydrating in mosses and lichen crusts (Bewley and Thorpe, 1974; Proctor et al., 2007; Kranner et al., 2008). Additionally, Maestre et al. (2013) showed a ~44% decrease in biocrust cover and lower net carbon fixation associated with a 2–3 degree temperature increase in biocrusts. Across the three seasons sampled in our study, moss crusts had the most fluctuation in respiration and net carbon fixation response. This high variability in gas exchange across seasons could indicate that moss crust communities are most sensitive to stress. On the other hand, cyanobacterial crusts had the most consistent gross and net carbon fixation rates across seasons while lichen crusts had the most consistent respiration rates, versus the most fluctuation in gross carbon fixation rates. It should be noted, however, that few differences were significant within crust types when comparing across seasons and incubation times, with the exception of respiration and net carbon fixation rates at 0.5 and 2 h incubations.

### 4.4. Recommendations for assessing carbon exchange of diverse biocrust types

Our results show that each biocrust type should be considered separately when assessing biogeochemical rates because each type can differ significantly from others in its maximum rates and responses to environmental stress. If studies document the makeup of biocrust cover at individual sites and link that with specific rates collected from those biocrust types, then primary factors modulating carbon exchange rates can be parameterized and carbon cycling models can be more accurately constructed. It is important to note that the amount of soil volume being used will affect carbon exchange measurements. Biocrusts are formed and maintained by photoautotrophs living at the soil surface to capture light and carbon dioxide, but they also live alongside a cohort of heterotrophs within the biocrust who are simultaneously respiring (Weber et al., 2022). The number of heterotrophs to be accounted for increases with soil volume being assessed by the apparatus being used in the study. Field based studies do not isolate the biocrusts when taking measurements, instead addressing the entire soil column and therefore capturing more respiration than is contributed by biocrust organisms. This means field assessment of biocrust photosynthesis is informative for the net carbon exchange of the soil column, while lab measurements can give a more accurate picture of the isolated effect of biocrust communities.

The amount of time the biocrust is wet and active before taking measurements must also be considered. In our study, the lab incubation time interval to the highest net fixation rate, differed in each season. Specifically, longer incubation times were associated with a longer dry period before sampling. For example, in summer 2020 most maximum rates were reached after 24 h, in 2021 after 12 h, and in 2022 after 36 h (though there was no 36 h interval for summer 2020). For the greatest probability of observing a net positive carbon fixation rate, one should wait at least 6–8 h for respiration rates to decrease. Waiting 24 h would be optimal but waiting 36 to 48 h would not be advised because cyanobacteria biomass by that point will begin to increase and estimates may start to drift from field conditions (Lange et al., 1992).

Additionally, when using the LI6400-XT, there is cross-sensitivity between relative humidity in the chamber and the CO_2_ reading. While there is a correction written into the equation calculating the CO_2_ reading, variation in the amount of positive/negative offset is machine specific. For our data, 2–4 machines were used at any one time to collect carbon flux values with replicates being systematically assigned to each machine so as to have an unbiased representation of biocrust type and incubation time. This mechanical issue should be taken into account when making carbon flux estimates and gauging how far from reality they may drift.

### 4.5. Conclusion

Our study provides one of the first comprehensive evaluations of biocrust carbon exchange from gypsum soils comparing five crust types and examining their carbon exchange response to time since rehydration and by season of sampling. All three factors, biocrust type, rehydration/incubation period, and sampling season were important in understanding carbon exchange in biocrusts at White Sands National Park, Chihuahuan Desert. Lichen and moss crusts had higher rates of gross and net carbon fixation than dark and light cyanobacterial crusts. The higher rate of carbon fixation in combination with the fragility of more structurally complex biocrust types (lichen and moss crusts) and their slow recovery rates (Kidron et al., 2020), highlight the importance of protecting biocrusts from destruction. Biocrust carbon fixation rates also varied with time since rehydration. After a watering event, a process of desiccation recovery is activated. Photosynthesis can recover within a short period of time while respiration remains high for an extended period. This makes comparisons of rates across studies somewhat challenging as there are a variety of pre-measurement incubation times used. Additionally, pre-sampling environmental conditions also have an impact upon the biogeochemical rates observed. The hot and dry conditions experienced by the biocrusts sampled in summer are noteworthy considering the likely consequences of climatic change. High temperatures and changes in precipitation patterns may diminish existing biocrust communities, especially mosses, which in our study were most sensitive to environmental stress. Quantifying and ground truthing the dynamics of biocrust carbon exchange will permit precise calibration of carbon cycling models and will thus enable us to better foresee impacts of global climate change on dryland carbon cycling.

## Data availability statement

The original contributions presented in the study are included in the article/Supplementary material, further inquiries can be directed to the corresponding authors. Data and R code associated with this manuscript are published to EDI Data Portal under the https://doi.org/10.6073/pasta/c6ffd88dc80df1ed1ec32ccdc477ac61.

## Author contributions

MH and NP led the project conception and design. DB provided site access and logistical support. MH collected and processed data with input from NP, AD-N, and LS. DJ provided statistical and R code advice. MH wrote the first manuscript draft with major edits provided by NP, subsequent edits provided by AD-N, LS, DJ, and DB. AD-N, LS, DJ, and NP approved manuscript submission. All authors contributed to the article and approved the submitted version.

## Funding

The research presented in this study was conducted under Permit: WHSA-2018-SCI-0012. This work was financially supported by White Sands National Park Service Grant no. P21AC11241-01 awarded to NP and the National Science Foundation: Drylands Critical Zone Network Grant no. EAR-2012475 awarded to AD-N and NP. In 2020 MH was awarded the Jornada LTER Graduate Student Annual Fellowship provided by funding from the National Science Foundation to New Mexico State University for the Jornada Basin Long-Term Ecological Research Program (Grant no. DEB 2025166).

## Conflict of interest

The authors declare that the research was conducted in the absence of any commercial or financial relationships that could be construed as a potential conflict of interest.

The handling editor VF declared a past collaboration with the author NP.

## Publisher’s note

All claims expressed in this article are solely those of the authors and do not necessarily represent those of their affiliated organizations, or those of the publisher, the editors and the reviewers. Any product that may be evaluated in this article, or claim that may be made by its manufacturer, is not guaranteed or endorsed by the publisher.
