# Supplementary figures and images for: Biocrust carbon exchange varies with crust type and time on Chihuahuan Desert gypsum soils
Source: Front Microbiol. 2023 May 10;14:1128631. doi: 10.3389/fmicb.2023.1128631 (PMC10208066; doi:10.3389/fmicb.2023.1128631)

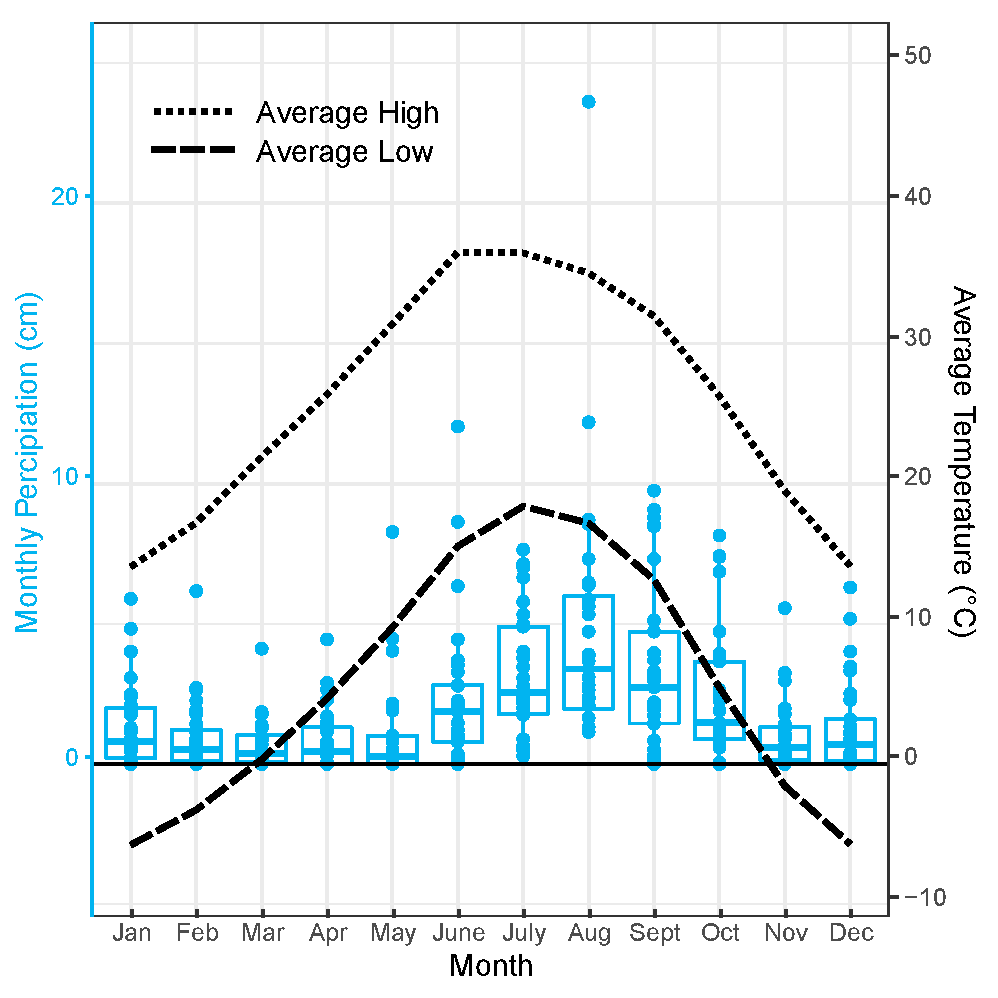

Supplement: Supplementary file 1 [file Image_1.TIFF]

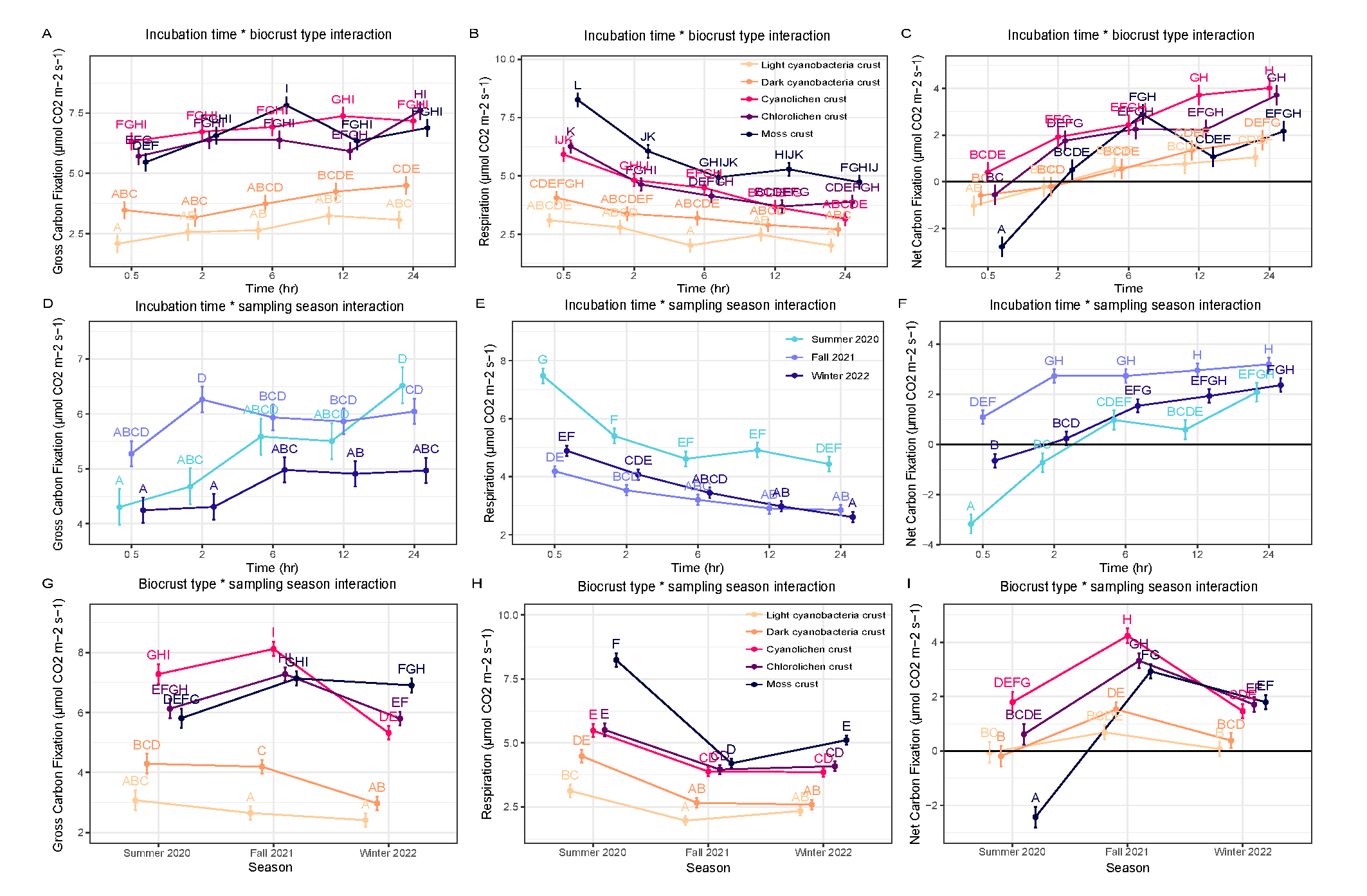

Supplement: Supplementary file 2 [file Image_2.TIFF]
