# Supplementary material for: Biocrust carbon exchange varies with crust type and time on Chihuahuan Desert gypsum soils
Source: Front Microbiol. 2023 May 10;14:1128631. doi: 10.3389/fmicb.2023.1128631 (PMC10208066; doi:10.3389/fmicb.2023.1128631)
Supplement: Supplementary file 3 [file Data_Sheet_1.docx]

Supplementary Material

**Biocrust Carbon Exchange Varies with Crust Type and Incubation Time across Three Seasons in Chihuahuan Desert**

**Mikaela Hoellrich*, Darren K. James, David Bustos, Anthony Darrouzet-Nardi, Louis Santiago, Nicole Pietrasiak^*^**

*** Correspondence:**Mikaela Hoellrich
[mhoellrich424@gmail.com](mailto:mhoellrich424@gmail.com)

Nicole Pietrasiak

[Nicole.Pietrasiak@unlv.edu](mailto:Nicole.Pietrasiak@unlv.edu)

# Supplementary data

Table 1. Table of coordinates corresponding to transect end points in the seasons they were collected.

| Season | Point 1 | Point 2 | Point 3 | Point 4 |
| --- | --- | --- | --- | --- |
| Summer 2020 | 32°43'47.49"N, 106°12'56.99"W | 32°43'46.58"N, 106°12'57.61"W | 32°43'47.15"N, 106°12'57.65"W | 32°43'46.79"N, 106°12'56.81"W |
| Fall 2021 | 32°43'46.36"N, 106°12'56.92"W | 32°43'46.17"N, 106°12'57.60"W | 32°43'45.48"N, 106°12'57.41"W | 32°43'45.68"N, 106°12'56.61"W |
| Winter 2022 | 32°43'47.19"N, 106°12'57.76"W | 32°43'46.22"N, 106°12'58.32"W | 32°43'46.91"N, 106°12'58.39"W | 32°43'46.40"N, 106°12'57.54"W |

Supplemental Table 2. High and low relative humidity at Holloman Air Force Base averaged across each month from 2012 to 2022.

| Month | DewPT_High | DewPT_Low | DewPT_Mean | High Relative Humidity (%) | Low Relative Humidity (%) | Mean Relative Humidity (%) |
| --- | --- | --- | --- | --- | --- | --- |
| 01 | -2.86 | -9.26 | -4.84 | 67.75 | 26.55 | 55.15 |
| 02 | -2.88 | -10.02 | -5.86 | 59.07 | 20.06 | 44.47 |
| 03 | -1.40 | -9.74 | -4.54 | 50.76 | 14.40 | 35.15 |
| 04 | -1.15 | -10.25 | -4.89 | 40.89 | 10.17 | 25.48 |
| 05 | 1.43 | -8.42 | -2.88 | 38.52 | 9.60 | 22.80 |
| 06 | 9.06 | -0.38 | 5.72 | 45.50 | 12.18 | 31.81 |
| 07 | 14.63 | 7.58 | 11.82 | 63.40 | 21.46 | 41.10 |
| 08 | 15.35 | 8.67 | 12.92 | 68.09 | 23.70 | 47.57 |
| 09 | 12.46 | 5.88 | 9.86 | 68.59 | 25.67 | 46.83 |
| 10 | 6.57 | -0.18 | 4.25 | 65.26 | 22.90 | 49.62 |
| 11 | 0.74 | -5.91 | -1.82 | 65.14 | 24.63 | 48.61 |
| 12 | -1.62 | -8.42 | -4.43 | 68.50 | 27.21 | 52.99 |

Table 3. Table of ANOVA results for gross fixation (A), respiration (B), and net fixation (C) across all three seasons, where *Sum Sq* is sum of squares, df is degrees of freedom, *F value* is the f statistic, and *Pr(>F)* is p-value associated with F statistic, and *Significance* is an indication of the degree of significance, *** is p>0.001, ** is p>0.01, and p>0.05.

| Source | Sum Sq | df | F value | Pr(>F) | Significance |
| --- | --- | --- | --- | --- | --- |
| A) Gross Fixation |  |  |  |  |  |
| (Intercept) | 15,211.48 | 1 | 5,540.73 | <0.001 | *** |
| Time | 159.93 | 4 | 14.56 | <0.001 | *** |
| Type | 1,636.86 | 4 | 149.06 | <0.001 | *** |
| Season | 187.08 | 2 | 34.07 | <0.001 | *** |
| Time:Type | 113.61 | 16 | 2.59 | 0.001 | *** |
| Time:Season | 83.51 | 8 | 3.80 | <0.001 | *** |
| Type:Season | 197.39 | 8 | 8.99 | <0.001 | *** |
| Time:Type:Season | 142.28 | 32 | 1.62 | 0.018 | * |
| Residuals | 1,509.97 | 550 |  |  |  |
| B) Respiration |  |  |  |  |  |
| (Intercept) | 9,053.55 | 1 | 5,639.31 | <0.001 | *** |
| Time | 248.60 | 4 | 38.71 | <0.001 | *** |
| Type | 712.29 | 4 | 110.92 | <0.001 | *** |
| Season | 289.52 | 2 | 90.17 | <0.001 | *** |
| Time:Type | 62.84 | 16 | 2.45 | 0.001 | ** |
| Time:Season | 24.56 | 8 | 1.91 | 0.056 |  |
| Type:Season | 60.18 | 8 | 4.69 | <0.001 | *** |
| Time:Type:Season | 74.66 | 32 | 1.45 | 0.054 |  |
| Residuals | 882.99 | 550 |  |  |  |
| C) Net Fixation |  |  |  |  |  |
| (Intercept) | 794.38 | 1 | 223.20 | <0.001 | *** |
| Time | 803.77 | 4 | 56.46 | <0.001 | *** |
| Type | 414.80 | 4 | 29.14 | <0.001 | *** |
| Season | 613.13 | 2 | 86.14 | <0.001 | *** |
| Time:Type | 190.93 | 16 | 3.35 | <0.001 | *** |
| Time:Season | 131.28 | 8 | 4.61 | <0.001 | *** |
| Type:Season | 302.19 | 8 | 10.61 | <0.001 | *** |
| Time:Type:Season | 194.53 | 32 | 1.71 | 0.01 | ** |
| Residuals | 1,957.43 | 550 |  |  |  |


[Figure 1]

Figure 1. Average monthly precipitation at White Sands National Park from 1992-2022 excluding those where there were more than 15 missing data points (National Weather Service 2023).
